# Supplementary material for: A yeast phenomic model for the influence of Warburg metabolism on genetic buffering of doxorubicin
Source: Cancer Metab. 2019 Oct 23;7:9. doi: 10.1186/s40170-019-0201-3 (PMC6806529; doi:10.1186/s40170-019-0201-3)
Supplement: Supplementary file 9 — Additional file 9. GO term-specific heatmaps for REMc/GTF-enriched clusters. GO term-specific heatmaps for significant GO process terms were generated as described in methods and Figs. 3 and 4. Any related child terms are presented in subsequent pages of the parent file name. GO terms with more than 100 children, with 2 or fewer genes annotated to the term, or a file size over 300KB are not shown. All heatmaps are generated with the same layout (see Figs. 3 and 4). [file 40170_2019_201_MOESM9_ESM.bz2 › Additional_File9_GOTermHeatmaps/Additional_File9_GOTermHeatmap/purine_ribonucleoside_triphosphate_metabolic_process.pdf]

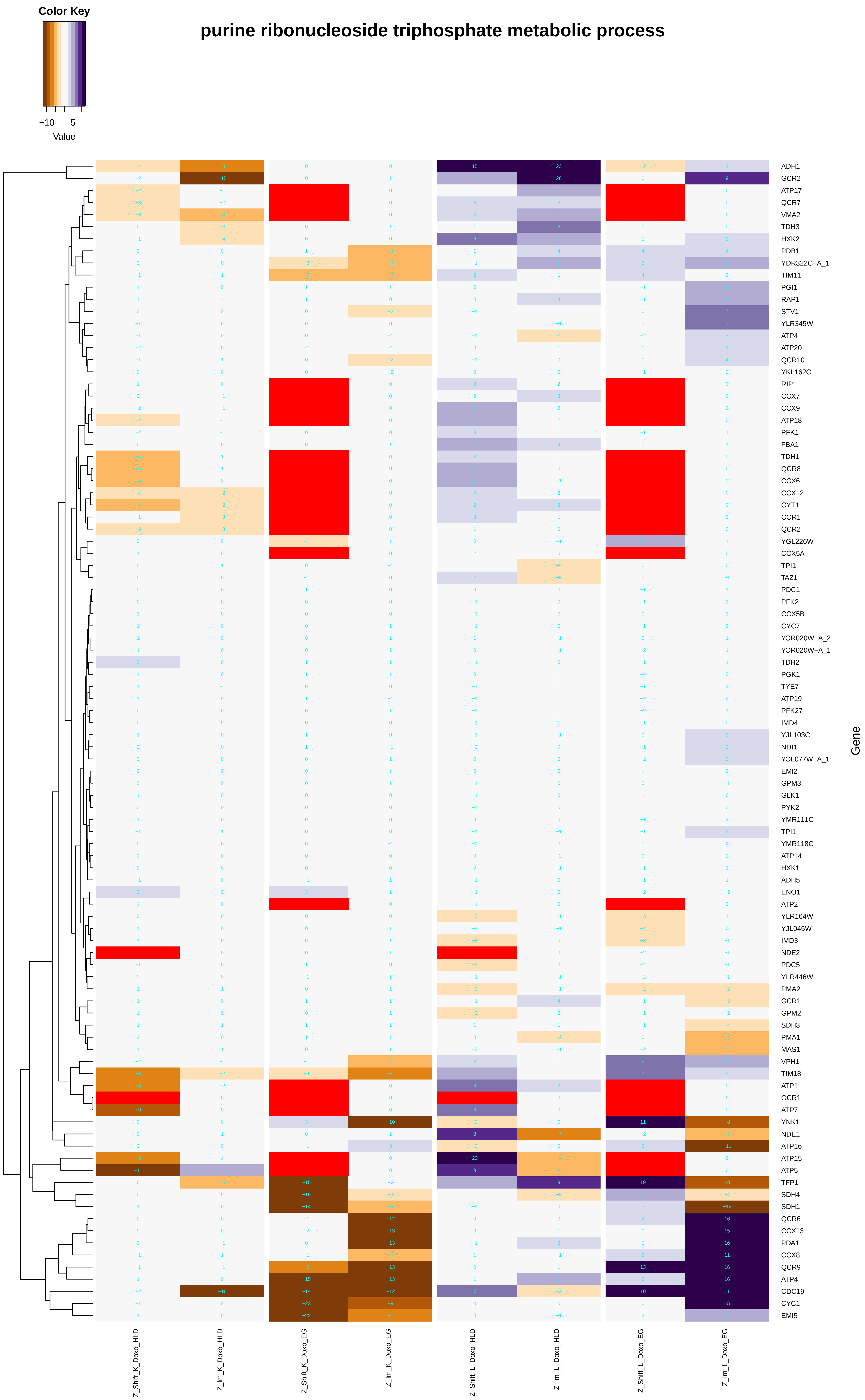

Color Key
−10
5
Value
purine ribonucleoside triphosphate metabolic process
ADH1
GCR2
ATP17
QCR7
VMA2
TDH3
HXK2
PDB1
YDR322C−A_1
TIM11
PGI1
RAP1
STV1
YLR345W
ATP4
ATP20
QCR10
YKL162C
RIP1
COX7
COX9
ATP18
PFK1
FBA1
TDH1
QCR8
COX6
COX12
CYT1
COR1
QCR2
YGL226W
COX5A
TPI1
TAZ1
PDC1
PFK2
COX5B
CYC7
YOR020W−A_2
YOR020W−A_1
TDH2
PGK1
TYE7
ATP19
PFK27
IMD4
YJL103C
NDI1
YOL077W−A_1
EMI2
GPM3
GLK1
PYK2
YMR111C
TPI1
YMR118C
ATP14
HXK1
ADH5
ENO1
ATP2
YLR164W
YJL045W
IMD3
NDE2
PDC5
YLR446W
PMA2
GCR1
GPM2
SDH3
PMA1
MAS1
VPH1
TIM18
ATP1
GCR1
ATP7
YNK1
NDE1
ATP16
ATP15
ATP5
TFP1
SDH4
SDH1
QCR6
COX13
PDA1
COX8
QCR9
ATP4
CDC19
CYC1
EMI5
Gene
Z_Shift_K_Doxo_HLD
Z_Im_K_Doxo_HLD
Z_Shift_K_Doxo_EG
Z_Im_K_Doxo_EG
Z_Shift_L_Doxo_HLD
Z_Im_L_Doxo_HLD
Z_Shift_L_Doxo_EG
Z_Im_L_Doxo_EG

purine ribonucleoside triphosphate biosynthetic process

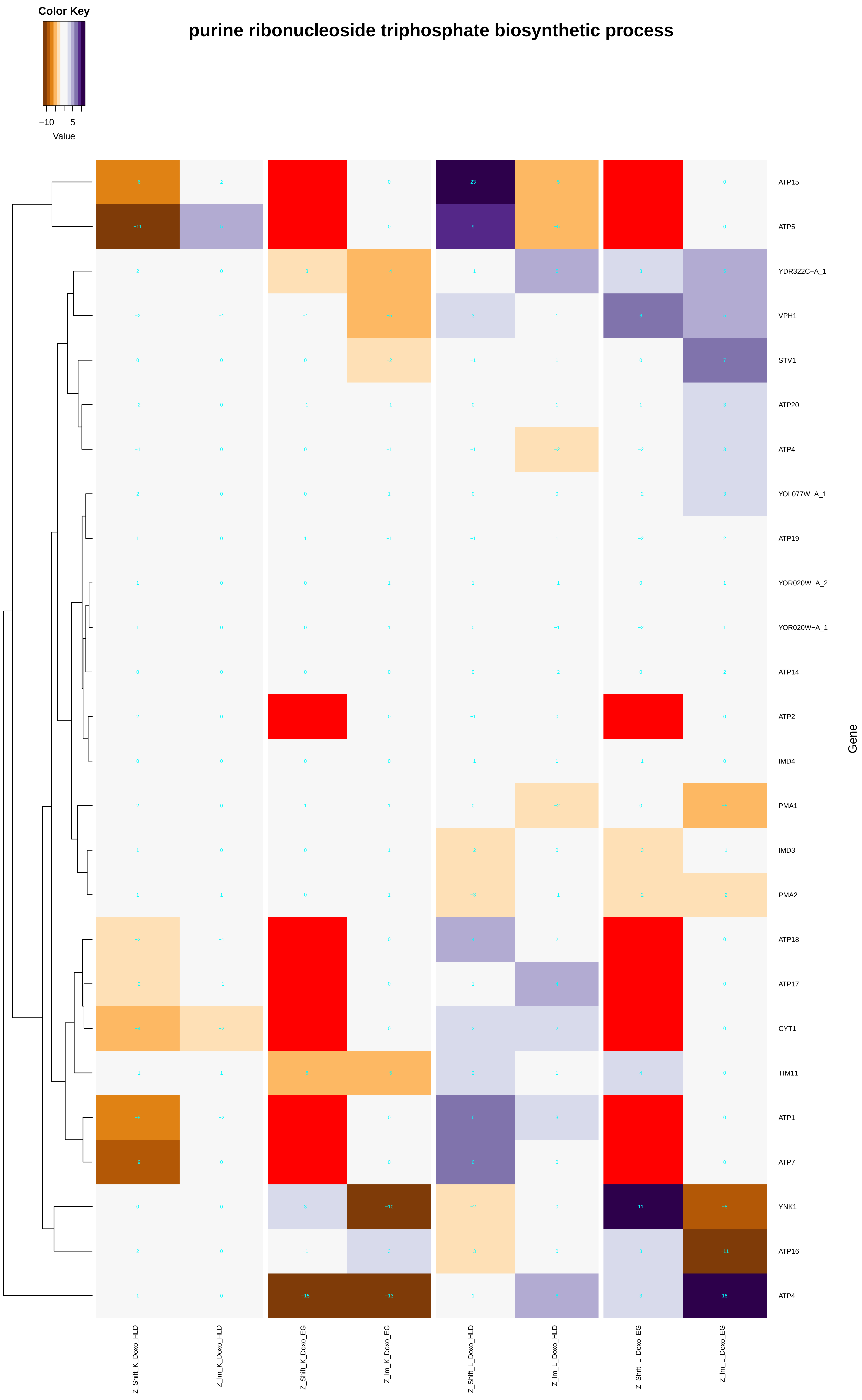

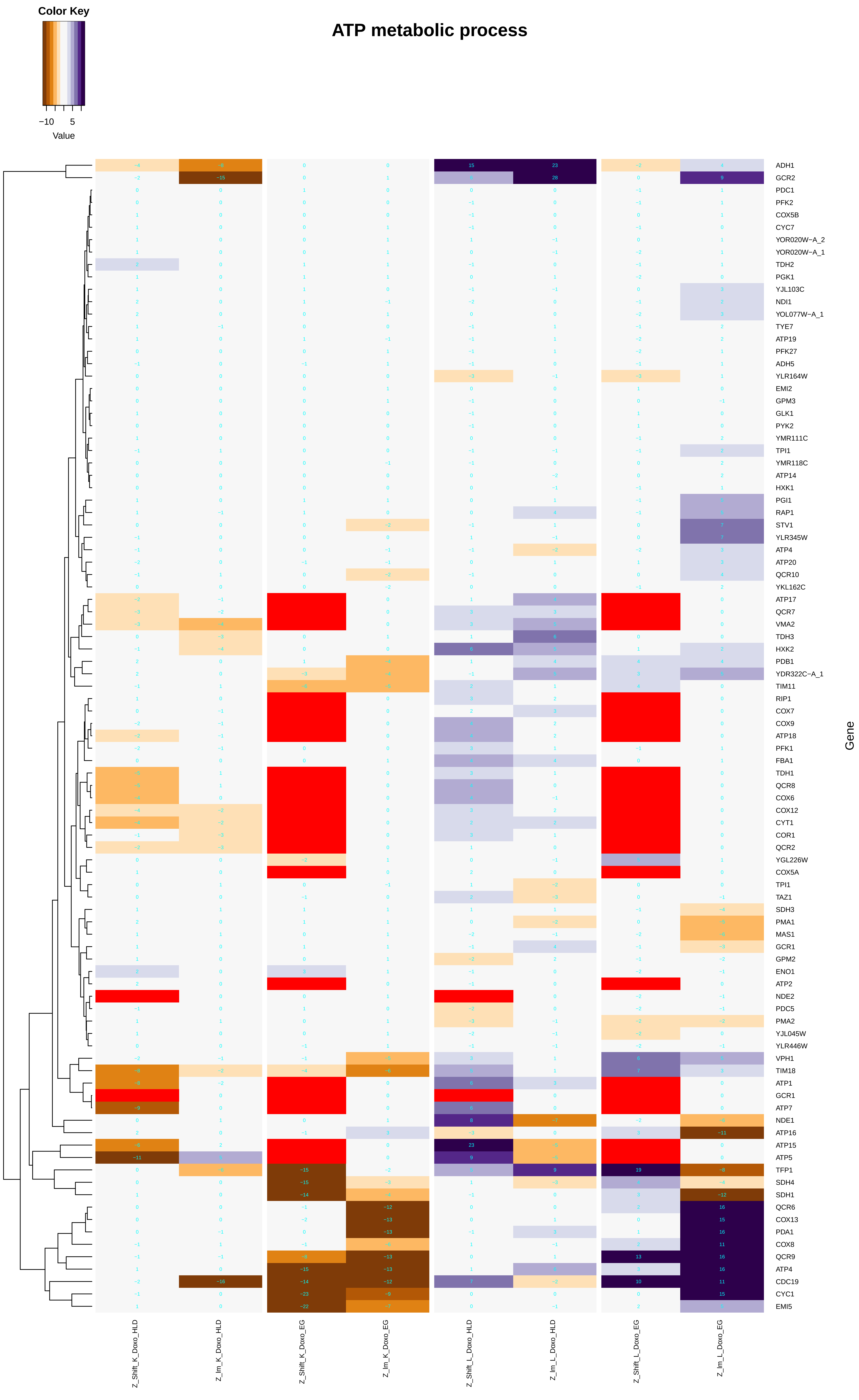

ATP metabolic process
Color Key
−10
5
Value
Gene
ADH1
GCR2
PDC1
PFK2
COX5B
CYC7
YOR020W−A_2
YOR020W−A_1
TDH2
PGK1
YJL103C
NDI1
YOL077W−A_1
TYE7
ATP19
PFK27
ADH5
YLR164W
EMI2
GPM3
GLK1
PYK2
YMR111C
TPI1
YMR118C
ATP14
HXK1
PGI1
RAP1
STV1
YLR345W
ATP4
ATP20
QCR10
YKL162C
ATP17
QCR7
VMA2
TDH3
HXK2
PDB1
YDR322C−A_1
TIM11
RIP1
COX7
COX9
ATP18
PFK1
FBA1
TDH1
QCR8
COX6
COX12
CYT1
COR1
QCR2
YGL226W
COX5A
TPI1
TAZ1
SDH3
PMA1
MAS1
GCR1
GPM2
ENO1
ATP2
NDE2
PDC5
PMA2
YJL045W
YLR446W
VPH1
TIM18
ATP1
GCR1
ATP7
NDE1
ATP16
ATP15
ATP5
TFP1
SDH4
SDH1
QCR6
COX13
PDA1
COX8
QCR9
ATP4
CDC19
CYC1
EMI5
Z_Shift_K_Doxo_HLD
Z_Im_K_Doxo_HLD
Z_Shift_K_Doxo_EG
Z_Im_K_Doxo_EG
Z_Shift_L_Doxo_HLD
Z_Im_L_Doxo_HLD
Z_Shift_L_Doxo_EG
Z_Im_L_Doxo_EG

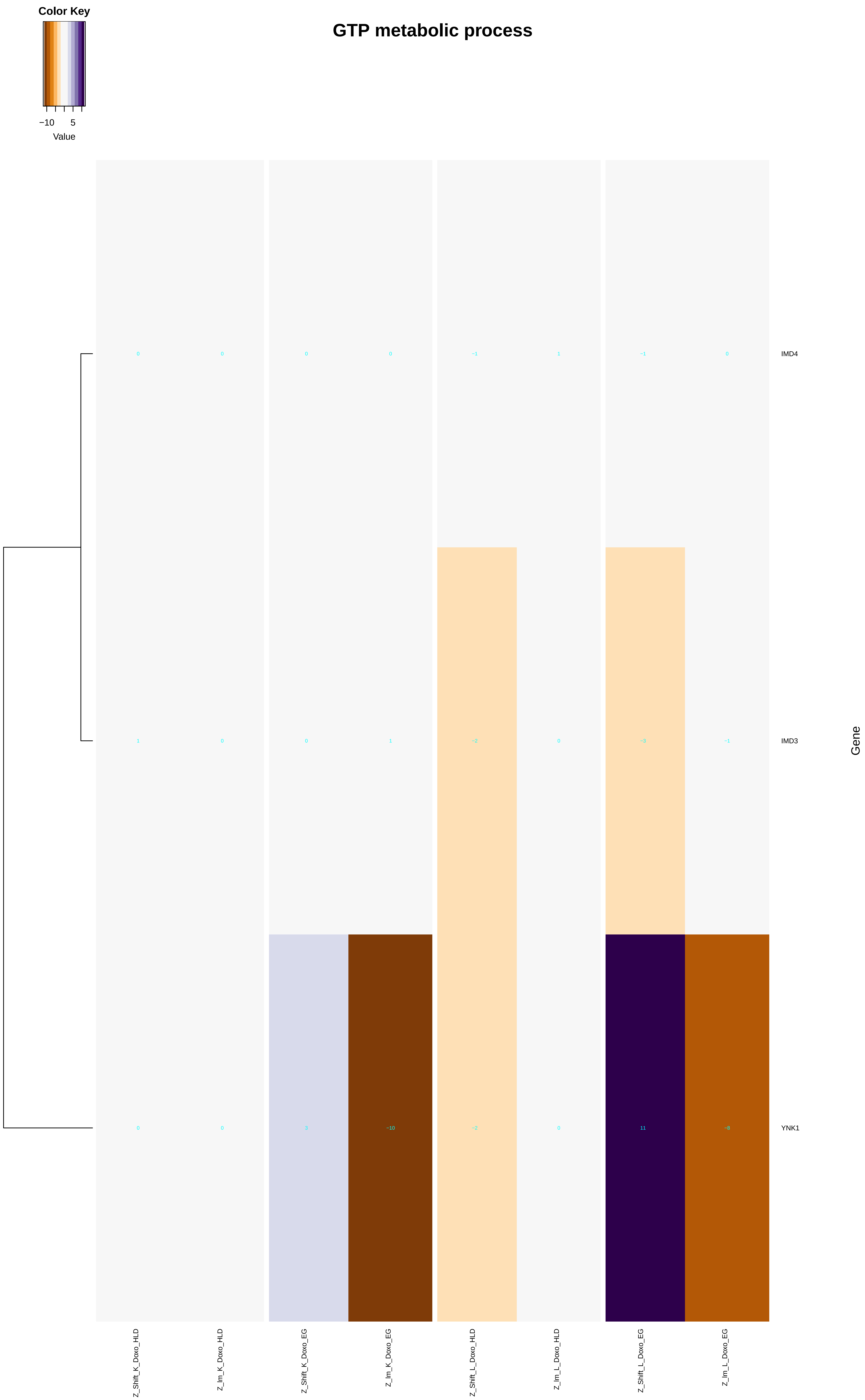
GTP metabolic process
Color Key
−10
5
Value
IMD4
IMD3
YNK1
Gene
0 0 0 0 −1 1 −1 0
1 0 0 1 −2 0 −3 −1
0 0 3 −10 −2 0 11 −8
Z_Shift_K_Doxo_HLD
Z_lm_K_Doxo_HLD
Z_Shift_K_Doxo_EG
Z_lm_K_Doxo_EG
Z_Shift_L_Doxo_HLD
Z_lm_L_Doxo_HLD
Z_Shift_L_Doxo_EG
Z_lm_L_Doxo_EG

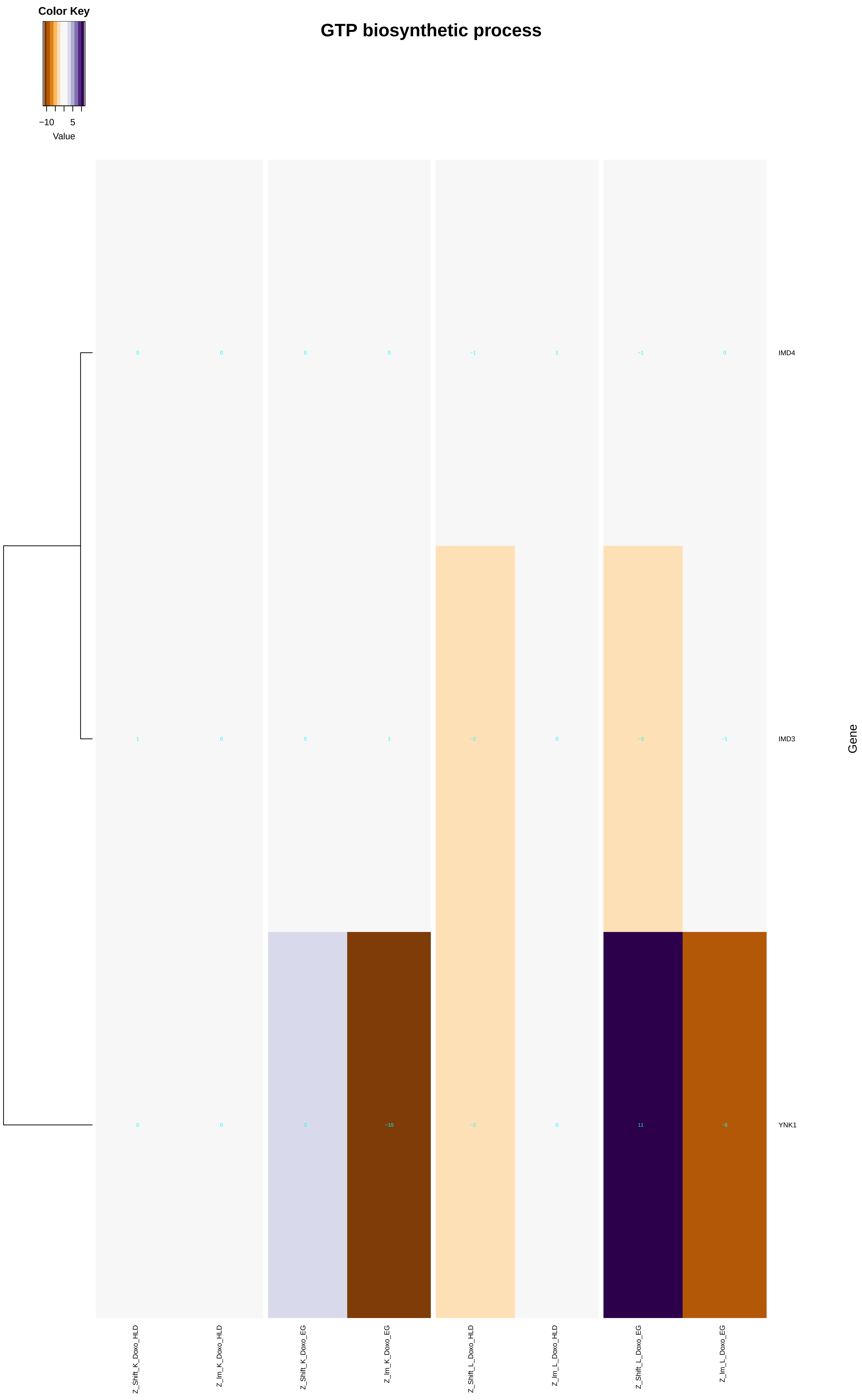
GTP biosynthetic process
Color Key
−10
5
Value
0
0
0
0
−1
1
−1
0
IMD4
1
0
0
1
−2
0
−3
−1
IMD3
0
0
3
−10
−2
0
11
−8
YNK1
Gene
Z_Shift_K_Doxo_HLD
Z_Im_K_Doxo_HLD
Z_Shift_K_Doxo_EG
Z_Im_K_Doxo_EG
Z_Shift_L_Doxo_HLD
Z_Im_L_Doxo_HLD
Z_Shift_L_Doxo_EG
Z_Im_L_Doxo_EG

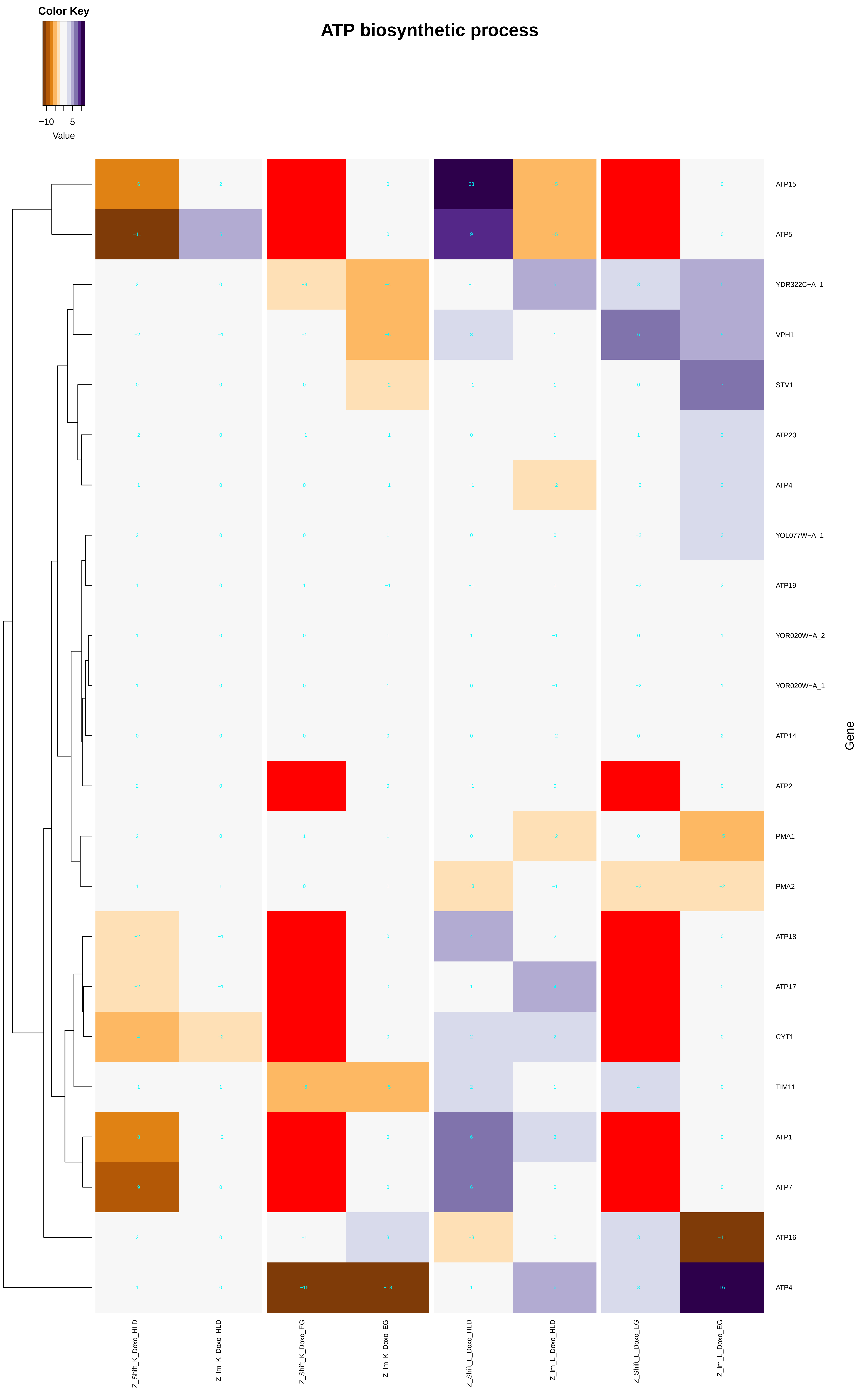
ATP biosynthetic process
Color Key
−10
5
Value
ATP15
ATP5
YDR322C−A_1
VPH1
STV1
ATP20
ATP4
YOL077W−A_1
ATP19
YOR020W−A_2
YOR020W−A_1
ATP14
ATP2
PMA1
PMA2
ATP18
ATP17
CYT1
TIM11
ATP1
ATP7
ATP16
ATP4
Gene
Z_Shift_K_Doxo_HLD
Z_Im_K_Doxo_HLD
Z_Shift_K_Doxo_EG
Z_Im_K_Doxo_EG
Z_Shift_L_Doxo_HLD
Z_Im_L_Doxo_HLD
Z_Shift_L_Doxo_EG
Z_Im_L_Doxo_EG

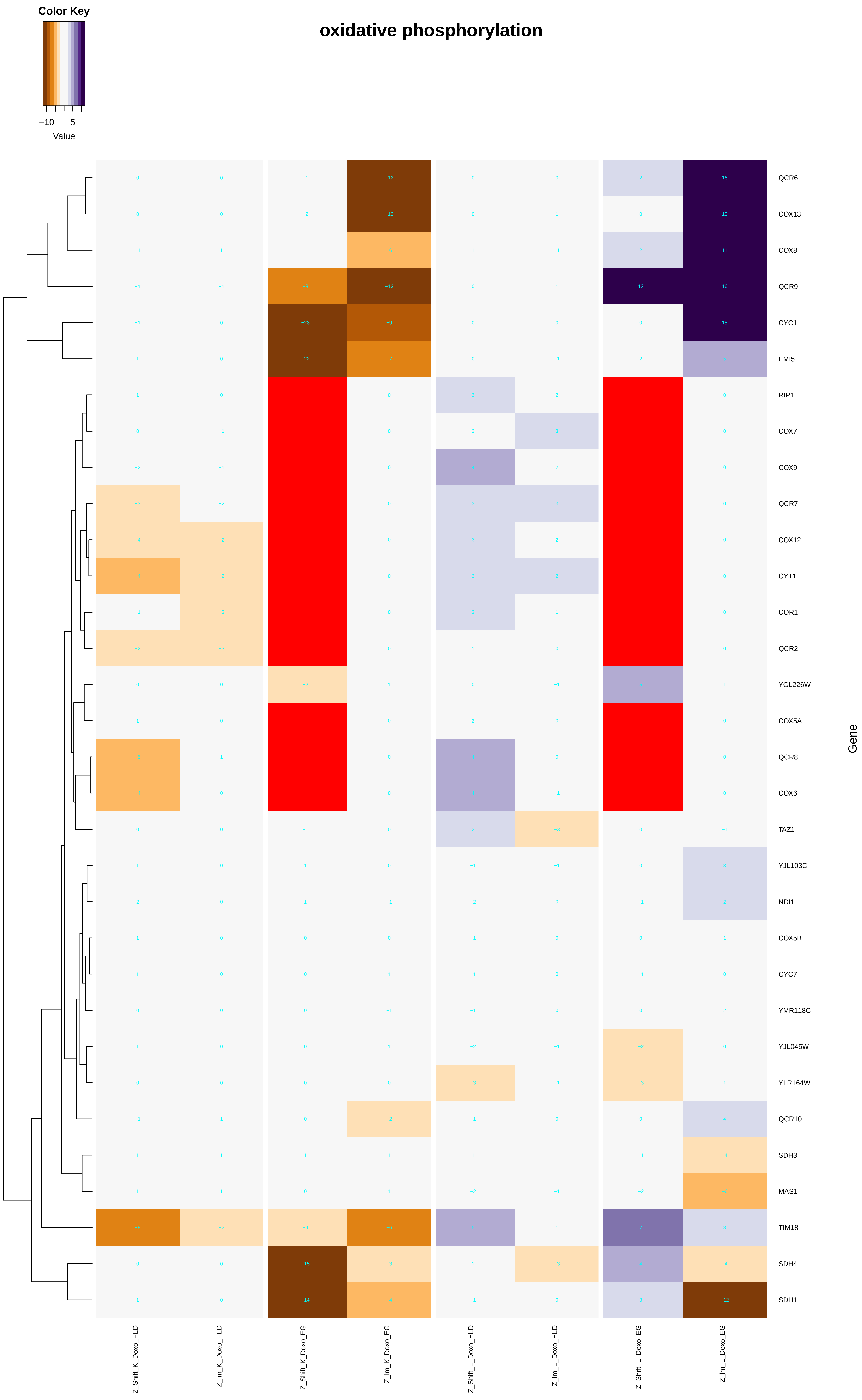
Color Key
−10
5
Value
oxidative phosphorylation
QCR6
COX13
COX8
QCR9
CYC1
EMI5
RIP1
COX7
COX9
QCR7
COX12
CYT1
COR1
QCR2
YGL226W
COX5A
QCR8
COX6
TAZ1
YJL103C
NDI1
COX5B
CYC7
YMR118C
YJL045W
YLR164W
QCR10
SDH3
MAS1
TIM18
SDH4
SDH1
Gene
Z_Shift_K_Doxo_HLD
Z_Im_K_Doxo_HLD
Z_Shift_K_Doxo_EG
Z_Im_K_Doxo_EG
Z_Shift_L_Doxo_HLD
Z_Im_L_Doxo_HLD
Z_Shift_L_Doxo_EG
Z_Im_L_Doxo_EG

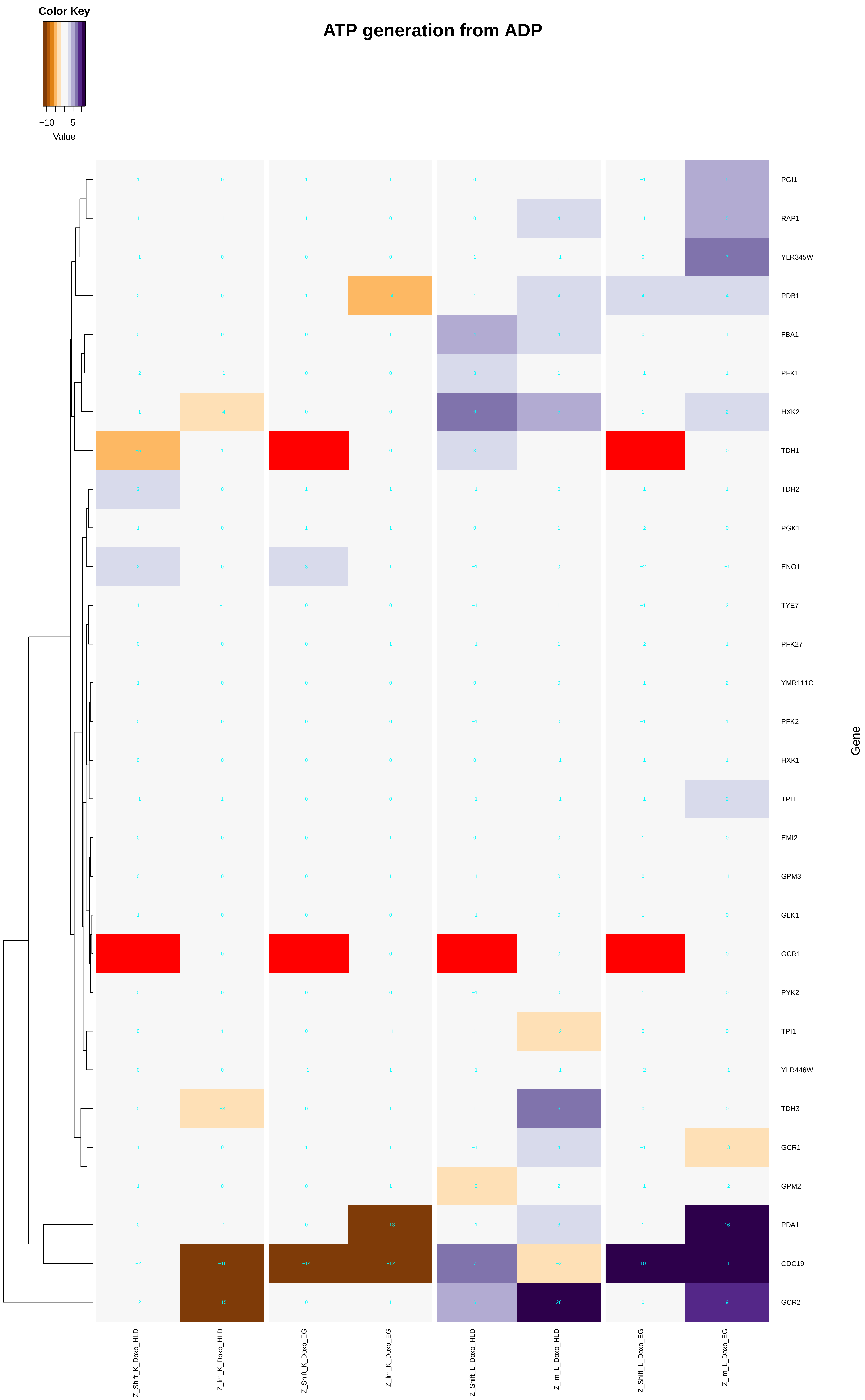
Color Key
−10
5
Value
ATP generation from ADP
PGI1
RAP1
YLR345W
PDB1
FBA1
PFK1
HXK2
TDH1
TDH2
PGK1
ENO1
TYE7
PFK27
YMR111C
PFK2
HXK1
TPI1
EMI2
GPM3
GLK1
GCR1
PYK2
TPI1
YLR446W
TDH3
GCR1
GPM2
PDA1
CDC19
GCR2
Gene
Z_Shift_K_Doxo_HLD
Z_Im_K_Doxo_HLD
Z_Shift_K_Doxo_EG
Z_Im_K_Doxo_EG
Z_Shift_L_Doxo_HLD
Z_Im_L_Doxo_HLD
Z_Shift_L_Doxo_EG
Z_Im_L_Doxo_EG

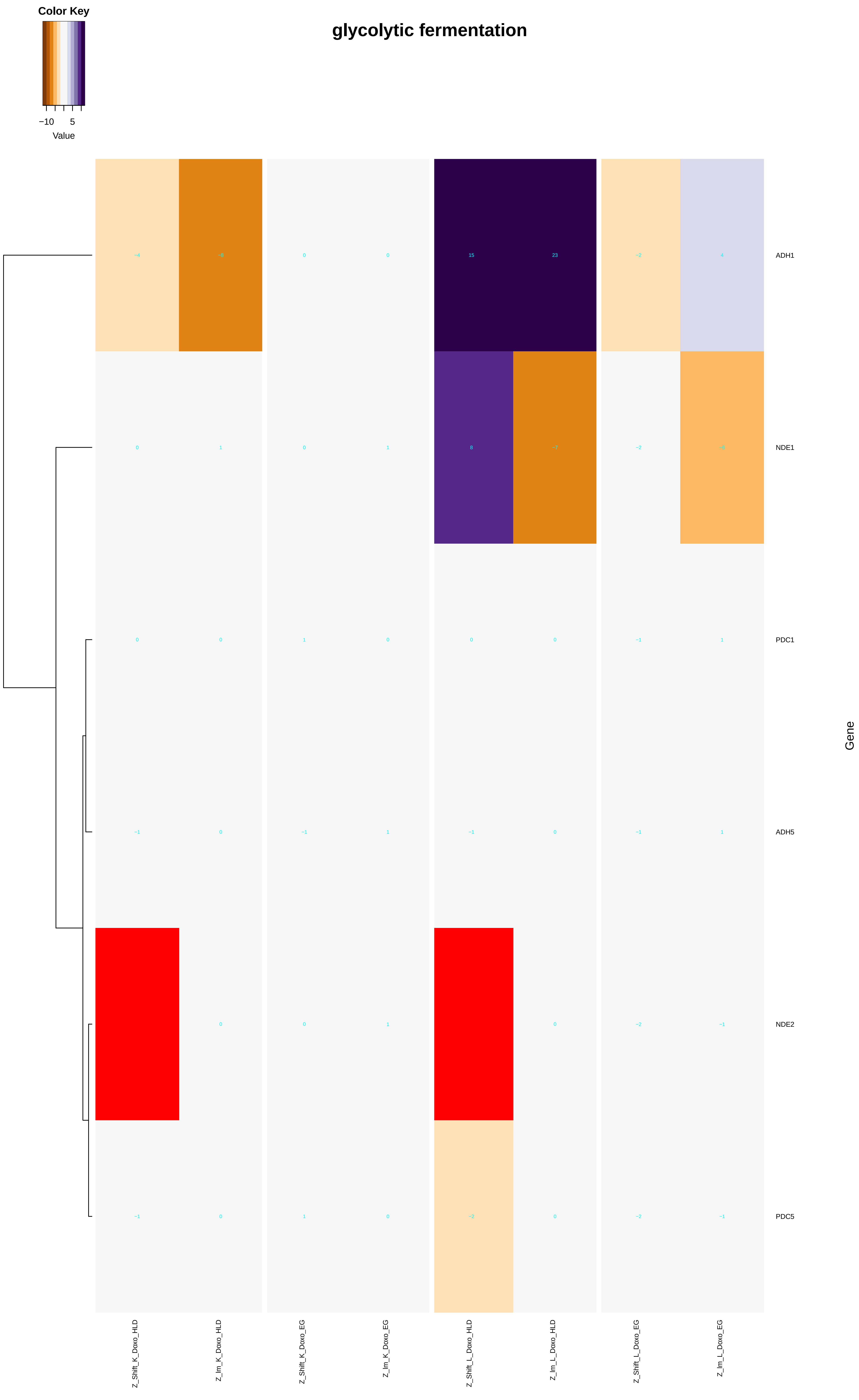
glycolytic fermentation
Color Key
−10
5
Value
ADH1
NDE1
PDC1
ADH5
NDE2
PDC5
Gene
Z_Shift_K_Doxo_HLD
Z_Im_K_Doxo_HLD
Z_Shift_K_Doxo_EG
Z_Im_K_Doxo_EG
Z_Shift_L_Doxo_HLD
Z_Im_L_Doxo_HLD
Z_Shift_L_Doxo_EG
Z_Im_L_Doxo_EG

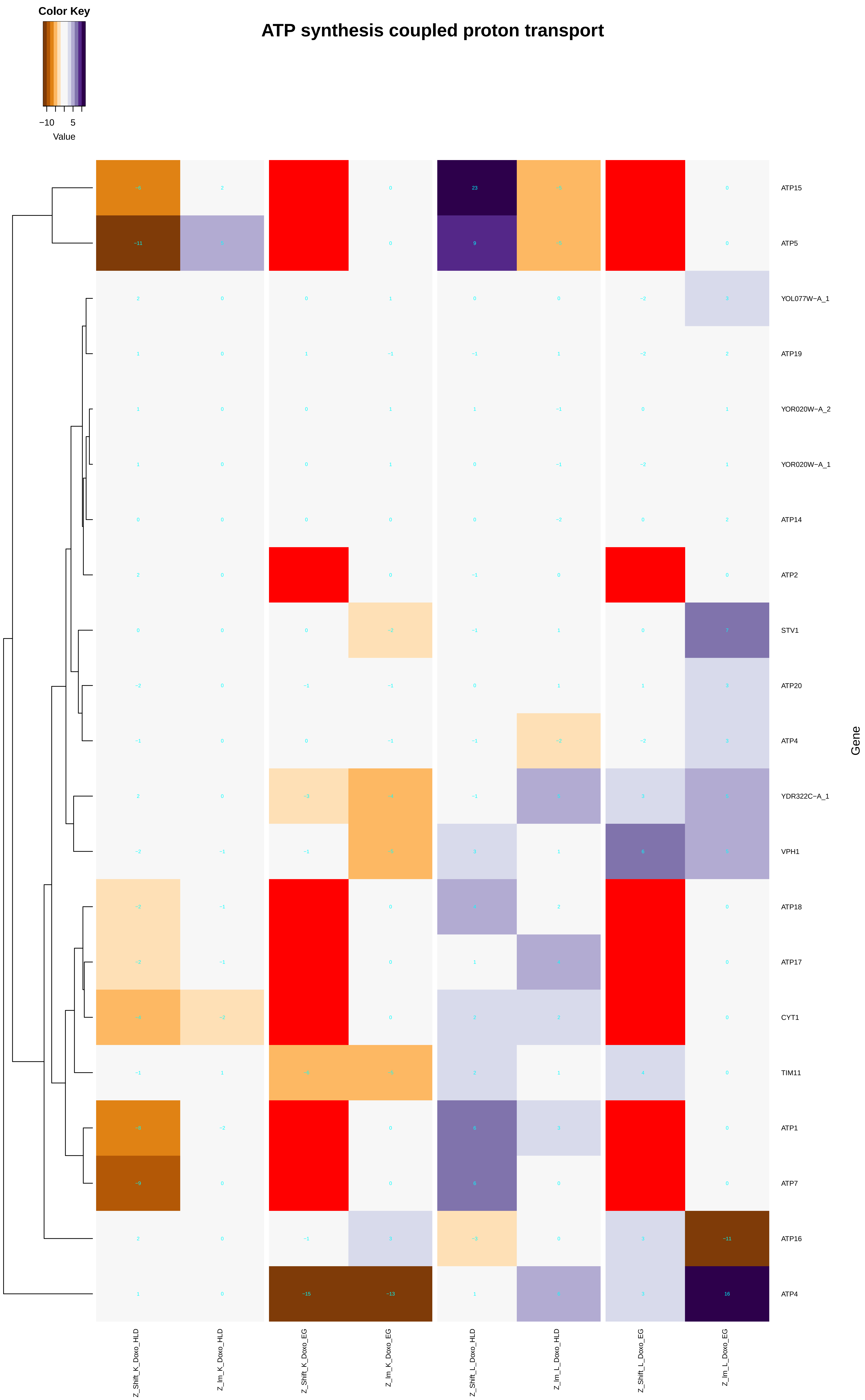
ATP synthesis coupled proton transport
Color Key
−10
5
Value
ATP15
ATP5
YOL077W−A_1
ATP19
YOR020W−A_2
YOR020W−A_1
ATP14
ATP2
STV1
ATP20
ATP4
YDR322C−A_1
VPH1
ATP18
ATP17
CYT1
TIM11
ATP1
ATP7
ATP16
ATP4
Gene
Z_Shift_K_Doxo_HLD
Z_Im_K_Doxo_HLD
Z_Shift_K_Doxo_EG
Z_Im_K_Doxo_EG
Z_Shift_L_Doxo_HLD
Z_Im_L_Doxo_HLD
Z_Shift_L_Doxo_EG
Z_Im_L_Doxo_EG

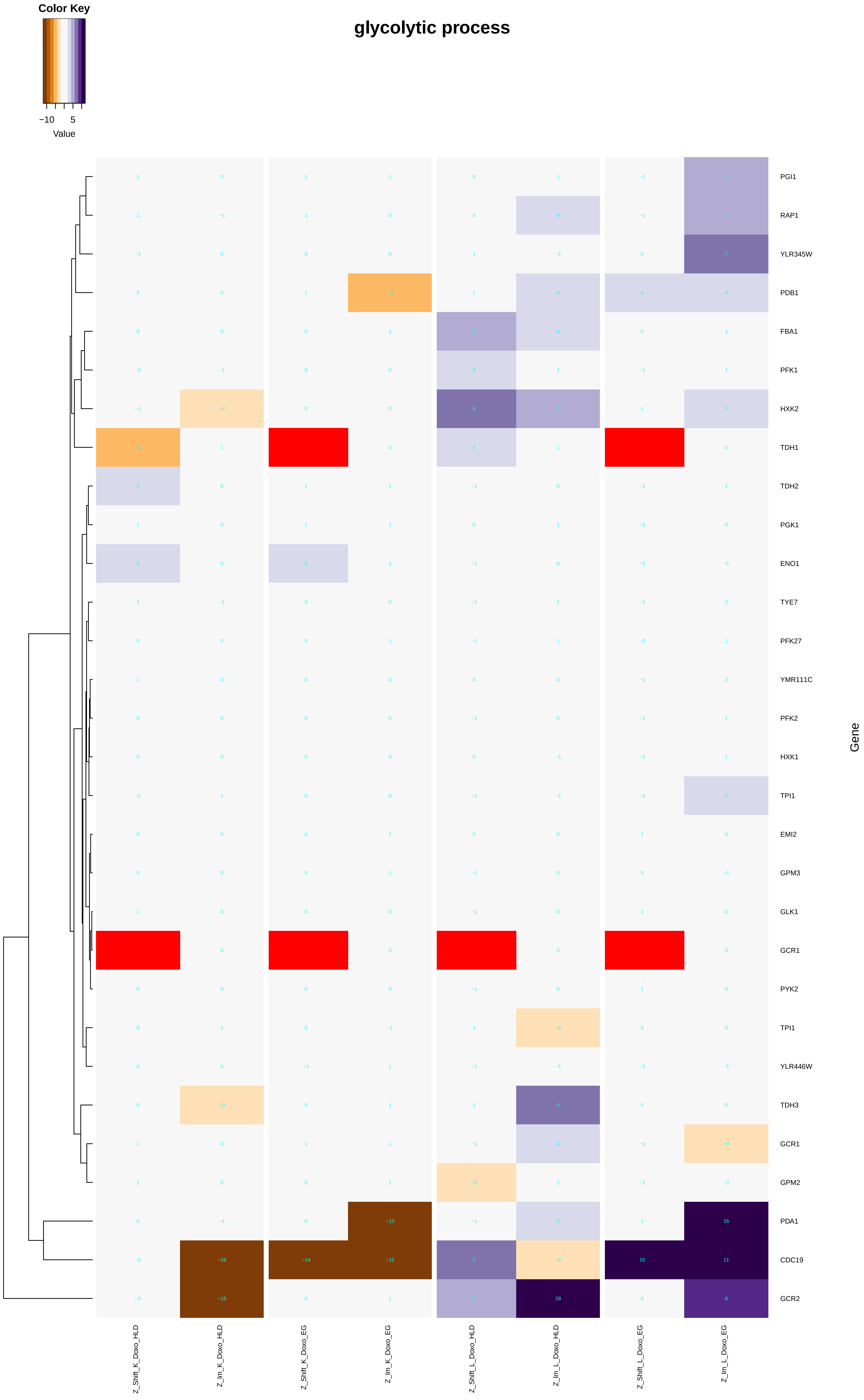
Color Key
−10
5
Value
glycolytic process
PGI1
RAP1
YLR345W
PDB1
FBA1
PFK1
HXK2
TDH1
TDH2
PGK1
ENO1
TYE7
PFK27
YMR111C
PFK2
HXK1
TPI1
EMI2
GPM3
GLK1
GCR1
PYK2
TPI1
YLR446W
TDH3
GCR1
GPM2
PDA1
CDC19
GCR2
Gene
Z_Shift_K_Doxo_HLD
Z_Im_K_Doxo_HLD
Z_Shift_K_Doxo_EG
Z_Im_K_Doxo_EG
Z_Shift_L_Doxo_HLD
Z_Im_L_Doxo_HLD
Z_Shift_L_Doxo_EG
Z_Im_L_Doxo_EG

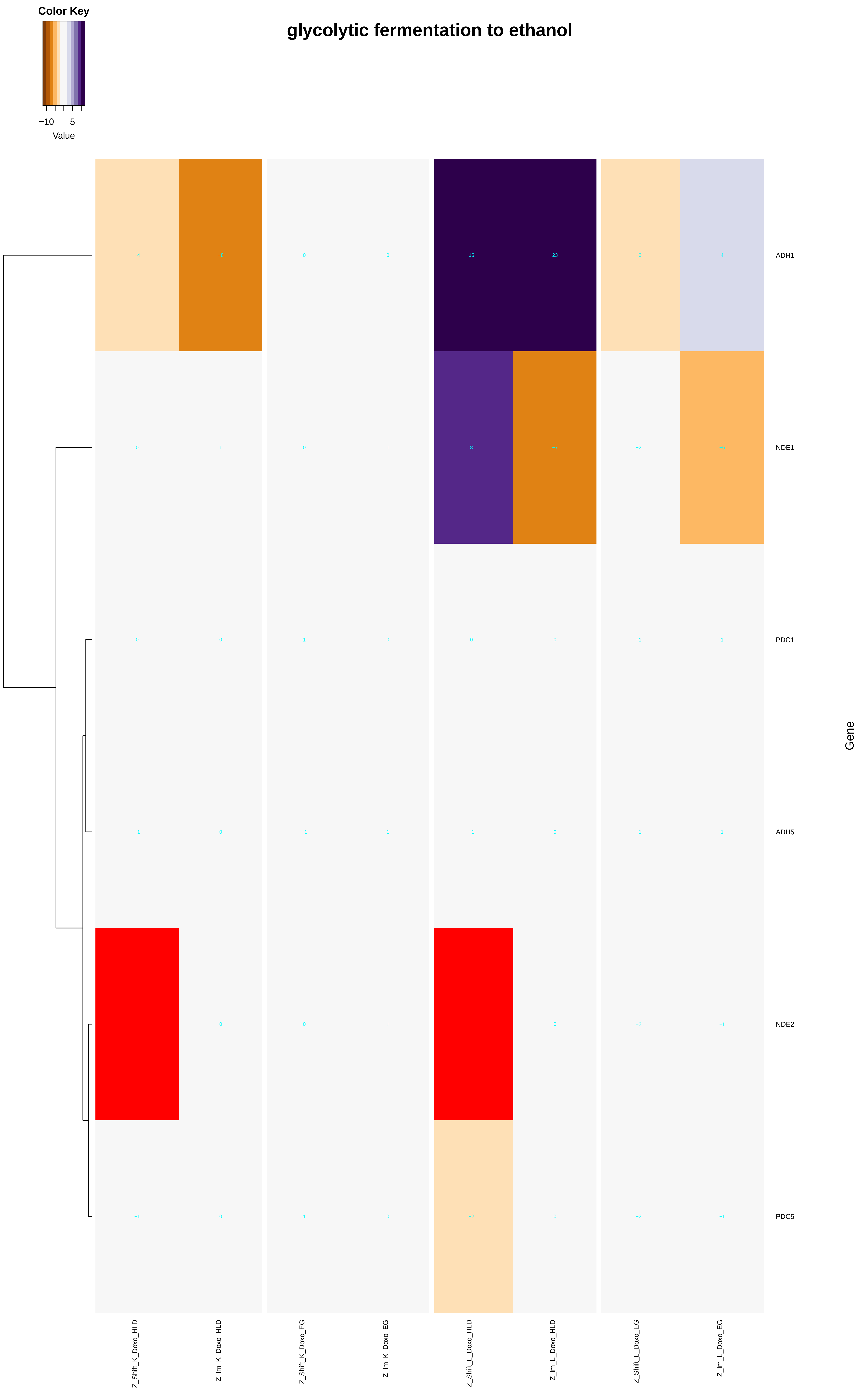
glycolytic fermentation to ethanol
Color Key
−10
5
Value
ADH1
NDE1
PDC1
ADH5
NDE2
PDC5
Gene
Z_Shift_K_Doxo_HLD
Z_Im_K_Doxo_HLD
Z_Shift_K_Doxo_EG
Z_Im_K_Doxo_EG
Z_Shift_L_Doxo_HLD
Z_Im_L_Doxo_HLD
Z_Shift_L_Doxo_EG
Z_Im_L_Doxo_EG
